# Manual acupuncture for the infertile female with polycystic ovary syndrome (PCOS): study protocol for a randomized sham-controlled trial

**DOI:** 10.1186/s13063-019-3667-y

**Published:** 2019-09-11

**Authors:** Qiao Wang, Haiping Deng, Ke Cheng, Zouqin Huang, Xiuqi Yin, Yichen Zhou, Yiqin Yang, Weidong Shen, Ling Zhao, Xueyong Shen

**Affiliations:** 1 Acupuncture & Tuina School, Shanghai University of Traditional Chinese Medicine, Shanghai, 201203 China; 2 Shanghai Pudong New District Hospital of Traditional Chinese Medicine, Shanghai, 201200 China; 3 Shuguang Hospital, Shanghai University of Traditional Chinese Medicine, Shanghai, 201203 China

**Keywords:** Polycystic ovary syndrome, Manual acupuncture, Traditional Chinese Medicine, Infertility, Herb medicine

## Abstract

**Background:**

Polycystic ovary syndrome (PCOS) is one of the most common endocrine diseases for women. Acupuncture is widely used for the infertile female because of it is non-invasive and has fewer side effects, but the powerful evidence for the clinic is still insufficient. Our study intends to explore the effect of manual acupuncture (MA) in the infertile female with PCOS.

**Methods:**

This study is a randomized, sham-controlled, patient-and assessor-blinded trial and aims to evaluate the effect of MA in women with PCOS and infertility. We will recruit 86 women aged 20–40 years with a diagnosis of infertility with PCOS. Participants will be randomly allocated in a 1:1 ratio to the MA group and the sham acupuncture (SA) group. Both groups will receive real herbal medicine treatment as a basic treatment twice a day for three menstrual cycles, the MA group receive real acupuncture treatment and the SA group received placebo acupuncture treatment (non-penetrating). All patients will receive acupuncture treatment twice per week for three menstrual cycles. The primary outcome is pregnancy rate and secondary outcomes include ovulation rate, sex hormones, insulin resistance index (IRI), PCOS symptoms, and Traditional Chinese Medicine (TCM) syndrome scores. Outcome measures will be collected at baseline, each menstrual cycle, the end of treatments, and six months after the last acupuncture treatment. The present protocol followed the SPIRIT guidelines and fulfilled the SPIRIT checklist.

**Discussion:**

This study will be conducted to compare the efficacy of MA versus SA. This trial will help to evaluate whether MA is effective in increasing pregnancy and ovulation rates of the infertile female with polycystic ovary syndrome.

**Trial registration:**

Chinese Clinical Trial Registry, ChiCTR1800014997. Registered on 27 February 2018.

**Electronic supplementary material:**

The online version of this article (10.1186/s13063-019-3667-y) contains supplementary material, which is available to authorized users.

## Background

Polycystic ovary syndrome (PCOS) is one of the most common endocrine diseases for women. Diagnosis is based on two of the following three findings: (1) oligo- and/or anovulation; (2) clinical and/or biochemical signs of hyperandrogenism; and (3) polycystic ovaries and exclusion of other etiologies [1]. The prevalence of PCOS under the Rotterdam criteria was 19.9% in 392 women aged 18–45 years [2]. It is one of the main causes of infertility in women during the reproductive period. PCOS causes > 75% of causes of anovulatory infertility [3].

Clomiphene citrate (CC) is the first-line treatment in anovulatory subfertility patients with PCOS. Gonadotrophins such as follicle-stimulating hormone (FSH) are, in addition to surgery, second-line treatments [4]. The cumulative ovulation rate of CC is as high as 90%, with a pregnancy rate of 50–70%, but the multiple pregnancy rate is up to 15% and rate of ovarian hyper-stimulation syndrome (OHSS) is 2% [5]. Meanwhile, it also has other impact effects [6] such as reversible ovarian enlargement (> 10%), visual symptoms, headaches, etc. Some studies [7, 8] reported that there were several associations between use of CC and birth defects.

Acupuncture and Traditional Chinese Medicine (TCM) methods have a long history of use for gynecological diseases [9] and the related research has increased in the past 20 years [10]. A prevalence study [11] reported that 29% of 428 infertile couples in eight community and academic infertility practices had used a complementary and alternative medicine (CAM) treatment for infertility, 22% had tried acupuncture, and 17% had tried herbal therapy. A review [12] investigated whether utility of TCM would be a feasible method to improve the outcome of female infertility treatment because of inadequate evidence. A meta-analysis [13] demonstrated that co-treatment with TCM and letrozole was more effective than letrozole monotherapy in the treatment of PCOS. Yanjing et al. [14] found that Chinese herbal formula combined with Electroacupuncture (EA) can remarkably improve the menstrual cycle, reduces body weight and the levels of luteinizing hormone (LH), LH/ follicle-stimulating hormone (FSH), total testosterone (T), anti-Mullerian hormone (AMH), improve ovulation, and pregnancy rates. We therefore selected Chinese herbal medicine as a basic treatment.

Recently, a scoping review [15] found that acupuncture had the highest level of evidence for its use among 12 different CAM methods in improving fertility outcomes although this evidence is inconclusive. Some evidence [16, 17] indicated that acupuncture was probably an effective treatments for PCOS, which can improve patients’ clinical symptoms, sex hormone levels, and menstrual cycle and effectively shorten reproduction cycles in infertility patients with PCOS. A systematic review [18] showed that acupuncture can be used to regulate menstruation, assist conception in women, and not increase the risk of multiple pregnancies, but the evidence is insufficient. There is a close relationship between the arrival of qi and therapeutic outcome. As *The Yellow Emperor*’*s Inner Classic: The Spiritual Pivot-The Nine Kinds of Needles and the Twelve Source Points* [19] states: “The most important thing in acupuncture is to get the acupuncture feeling, when it appears, the curative effect will appear.” We therefore use manual acupuncture (MA) to obtain the qi sensation (acupuncture feeling) to get the curative effect.

Nevertheless, Pastore et al. [20] also claimed that both true acupuncture (TA) and sham acupuncture (SA) had a similar improvement in their LH/FSH ratio for these women with PCOS. A randomized clinical trial [21] did not support acupuncture as an infertility treatment in such women. The conclusion of the multicenter-large sample study [22] was potentially incorrect because it had serious flaws. Indeed, the evidence for the effect of acupuncture in the treatment for infertile women with PCOS is still insufficient. Therefore, this study aims to investigate the clinical effectiveness of acupuncture in infertile women with PCOS. Our hypothesis is that MA will regulate the menstrual cycle and improve the ovulation and pregnancy rates.

## Methods/design

### Study design

**Fig. 1:**
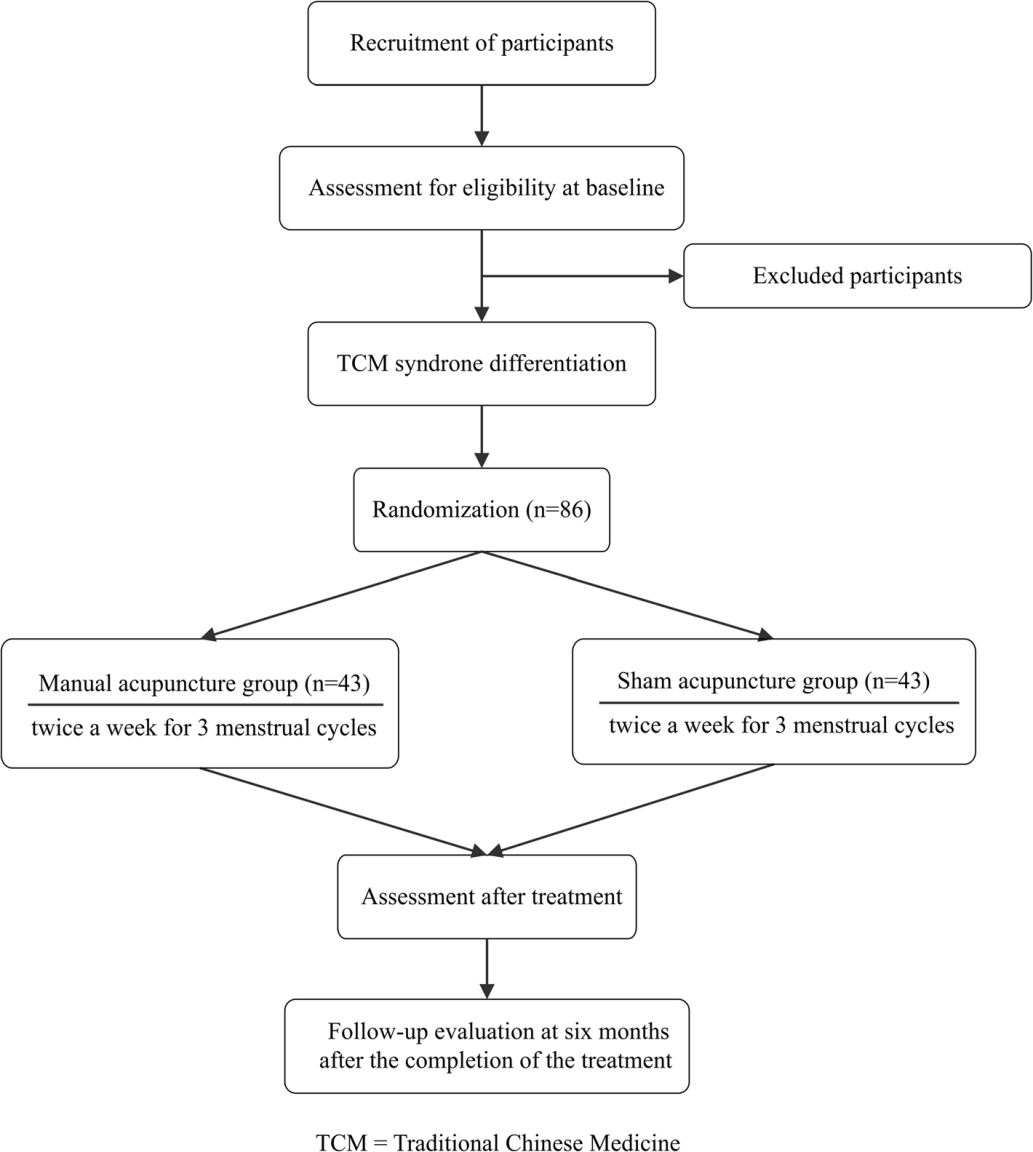
Study flow chart

This is a randomized, sham-controlled, patient-and assessor-blinded trial. A target sample of 86 participants will be recruited from the gynecology clinic at Shuguang Hospital. The flow chart is shown in Fig. 1. The present protocol followed the SPIRIT guidelines and fulfilled the SPIRIT checklist (Additional file 1). The trial protocol is in accordance with the principles of the Declaration of Helsinki and has been approved by Institution review board (IRB) of Shuguang Hospital (the Affiliated Hospital of Shanghai University of TCM) (approval no. 2017–569–52-01). This trial was registered at the Chinese Clinical Trial Registry (ChiCTR1800014997). Any changes that need to be made in the trial protocol will be communicated to all investigators, the ethics committees, and the trial registry. Written informed consent will be obtained from each participant.

### Sample size, randomization, and blinding

Due to the lack of sufficient preliminary studies, it is difficult to determine adequate sample size. According to the studies [23, 24], we hypothesize that the pregnancy rate after MA is 76% and the pregnancy rate after SA is 40%. The calculation equation we used was α = 0.05, 1-β = 0.80 and we determined that a sample size of 36 patients in each group would be sufficient to detect the statistical difference between the two groups, allowing for a 20% withdrawal rate. We will plan to enroll a total of 86 participants with 43 participants in each group. According to the random number sequence generated by Excel, the patients will be randomly divided into either the MA group or the SA group in a 1:1 ratio. We set the blind code in the case for remaining blind when the patients have the adverse effects. The random code and blind code will be conducted by a “third party” independent of the study using opaque envelopes. The envelopes will be sealed and shuffled and the assignment records will not be disclosed until the end of the study. Trial participants, formula doctor, outcome assessors, and data analysts will be blinded to the treatment allocation to minimize potential sources of bias. Because of the special nature of acupuncture, only acupuncturists will not be blinded to the treatment allocation.

### Inclusion criteria

Participants who meet all the following requirements will be allowed for enrollment:

1. Women aged 20–40 years.
2. Patients who meet the diagnostic criteria for both infertility and PCOS:
  - Patients who meet the diagnosis of PCOS defined by the Rotterdam criteria proposed in 2003 [1]:
    Oligomenorrhea or amenorrhea: oligomenorrhoea is defined as an intermenstrual interval > 35 days or < 8 menstrual bleedings in the past year. Amenorrhea is defined as complete cessation of menstrual cycles for six months or more when a patient has previously had regular cycles and for 12 months or more when the patient has had irregular cycles;
    Clinical or biochemical hyperandrogenism: biochemical hyperandrogenemia is defined as a total serum testosterone concentration > 60 ng/ dL and clinical hyperandrogenism is defined as a Ferriman-Gallwey (FG) score ≥ 5 in mainland China;
    Polycystic ovary morphology: this is defined as ≥ 12 antral follicles (2–9 mm in diameter) or an ovarian volume > 10 mL on transvaginal scanning;
  - Patients who meet the diagnosis of infertility [25]: women who fail to achieve a clinical pregnancy after 12 months or more of regular unprotected sexual intercourse; this includes secondary infertility which is failure to conceive after a previous pregnancy.
3. Husband’s semen examination is normal.
4. Willingness to sign the consent form.

### Exclusion criteria

Participants meeting any of the following criteria will be excluded:

1. Hyperprolactinemia, adrenal or ovarian tumors secreting abnormal increase of androgen, thyroid disease, suspected Cushing syndrome, and other heart and kidney diseases.
2. Those who had used hormone drugs such as estrogen or oral contraceptives in the last month.
3. Those who had used other drugs that affect reproductive function or metabolism (such as anti-obesity drugs, anti-diabetic drugs, etc.) or have participated in other clinical trials in the past two months.
4. Those who had received acupuncture treatment.
5. Those who had other infertility factors (such as tubal blockage, immune infertility, etc.).
6. Those who were unable to cooperate with the completion of the study, including patients with infectious diseases, mental illness, and other medical histories.

### Interventions

Both TA and SA groups will receive acupuncture sessions for a total of three menstrual cycles. All the patients will receive herbal medicine twice a day for three menstrual cycles. The same doctor (the professor of obstetrics and gynecology in Shuguang hospital who has 35 years of work experience) will slightly adjust the formula each week depending on the changes in the patients’ symptoms, tongue coating, and pulse (main herbal formula components are shown in Table 1). During the menstrual period, patients will be orally administered the *Taohong Siwu* decoction. Non-menstrual period treatment will be based on pattern differentiation: (1) spleen–kidney Yang deficiency will be given *Bushen Tiaojing* decoction; and (2) liver–kidney Yin deficiency will be given *Guishao Dihuang* decoction. Participants will receive all herbs from the same hospital and will be instructed on how to make the decoction.

**Table 1:** Main herbal formula selection

Period	Pattern	Formula	Composition
Menstrual period		*Taohong Siwu* decoction	*Radix Rehmanniae* Preparata, *Radix Angelicae Sinensis*, *Radix Paeoniae Alba*, *Rhizoma Chuanxiong*, *Semen Persicae*, *Flos Carthami*
Non-menstrual period	Spleen–kidney Yang deficiency	*Bushen Tiaojing* decoction	*Radix Angelicae Sinensis*, *Rhizoma Chuanxiong*, *Radix Rehmanniae Preparata*, *Radix Paeoniae Alba*, *Poria*, *Radix Salviae Miltiorrhizae*, *Semen Cuscutae*, *Radix Astragali*, *Fructus Lycii*, *Rhizoma Dioscoreae*
	Liver–kidney Yin deficiency	*Guishao Dihuang* decoction	*Radix Rehmanniae*, *Radix Paeoniae Alba*, *Fructus Lycii*, *Cortex Moutan*, *Rhizoma Anemarrhenae*, *Radix Ginseng*, *Radix Glycyrrhizae*, *Cortex Lycii*

**Fig. 2:**
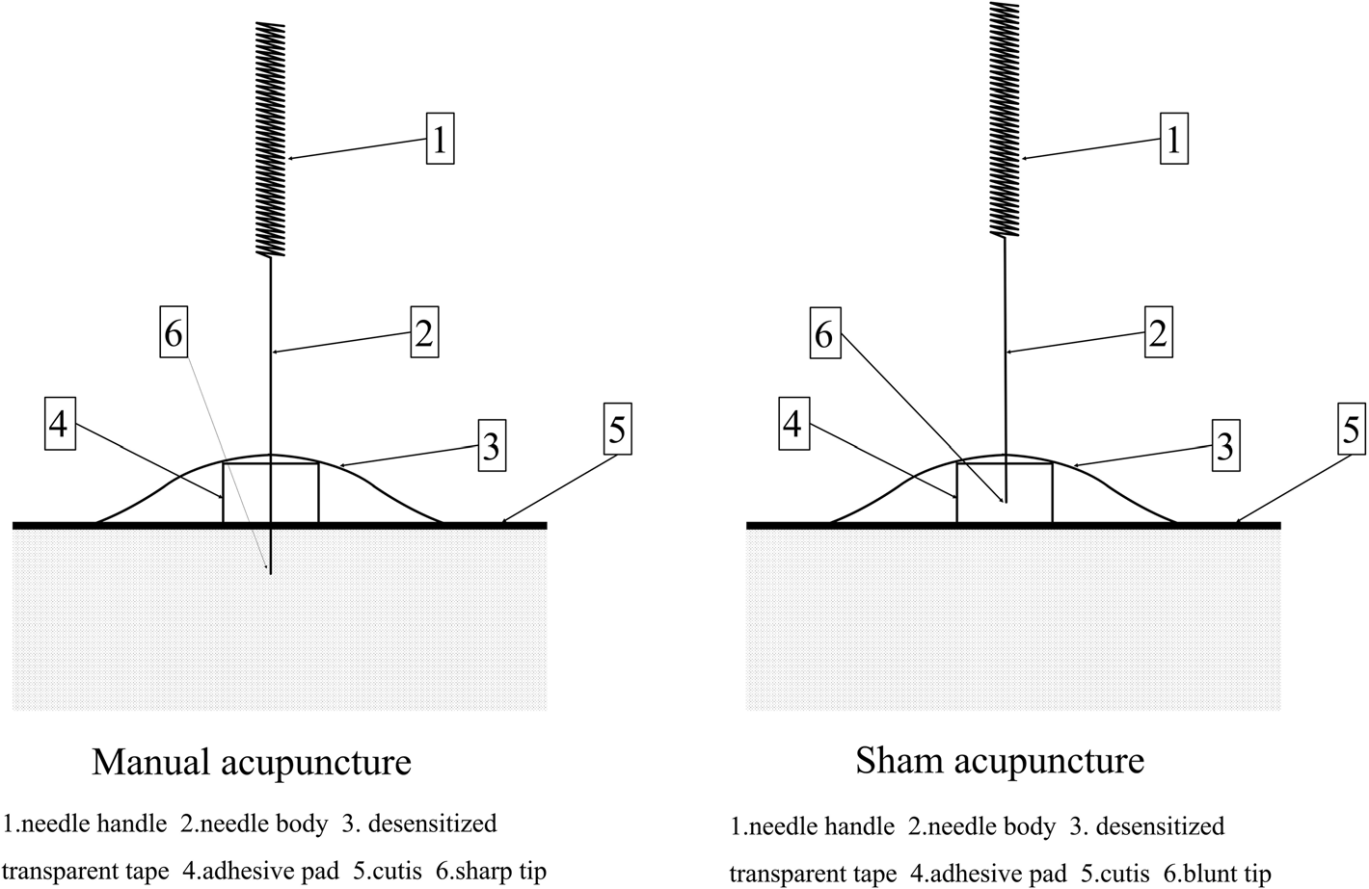
Schematic diagram of acupuncture device

According to the textbook *Acupuncture and Moxibustion* [26], and taking opinions from Chinese acupuncture experts, the essential acupuncture points selected are as follows: RN4 (*Guanyuan*); EX-CA1 (*Zigong*); ST29 (*Guilai*); ST36 (*Zusanli*); and SP6 (*Sanyinjiao*). The additional individualized acupuncture points will be chosen by the practitioners according to the patterns of identification: (1) spleen–kidney Yang deficiency plus RN6 (*Qihai*), RN12 (*Zhongwan*), ST25 (*Tianshu*); and (2) liver–kidney Yin deficiency will add with bilateral KI3 (*Taixi*), KI6 (*Zhaohai*). According to the menstrual cycle, the follicular phase will use the acupuncture points bilateral KI3 (*Taixi*) and KI6 (*Zhaohai*); the ovulatory phase will use the acupuncture points bilateral LR3 (*Taichong*), SP10 (*Xuehai*), PC6 (*Neiguan*), and DU20 (*Baihui*) (Table 2). A placebo device [27] will be applied in both groups for better implementation of blindness. The schematic diagram of acupuncture treatment is shown in Fig. 2. The acupuncture treatment in both groups will take 30 min per session, twice per week, from the third day of menstruation to the third day after ovulation for three menstrual cycles. Acupuncture treatments will be stopped upon a positive pregnancy test. The test will be done on the 14th day after each ovulation.

**Table 2:** Acupuncture point selection

Point	Location
RN4 (*Guanyuan*)	On the lower abdomen at the anterior midline, 3 *cun* below the umbilicus
EX-CA1^a^ (*Zigong*)	On the lower abdomen, 4 *cun* below the center of the umbilicus and 3 *cun* lateral to the anterior midline
ST29^a^ (*Guilai*)	1 *cun* cranial to the pubic bone and 2 *cun* lateral of the midline
ST36^a^ (*Zusanli*)	At the anterior aspect of the leg 3 *cun* inferior to ST35 (*Dubi*) on the line connecting ST35 (*Dubi*) to ST41 (*Jiexi*)
SP6^a^ (*Sanyinjiao*)	On the tibial aspect of the leg posterior to the medial border of the tibia, 3 *cun* superior to the prominence of the medial malleolus
Syndrome 1	
RN6 (*Qihai*)	With the patient supine, the point is halfway between the center of the umbilicus and RN4 (G*uan yuan*)
RN12 (*Zhongwan*)	On the upper abdomen at the anterior median line, 4 *cun* above the umbilicus
ST25^a^ (*Tianshu*)	On the upper abdomen 2 *cun* lateral to the center of the umbilicus
Syndrome 2 and phase 1	
KI3^a^ (*Taixi*)	On the posteromedial aspect of the ankle in the depression between the prominence of the medial malleolus and the calcaneal tendon
KI6^a^ (*Zhaohai*)	On the medial aspect of the foot in the depression inferior to the medial malleolus, 1 *cun* inferior to the prominence of the medial malleolus
Phase 2	
LV3^a^ (*Taichong*)	On the dorsum of the foot between the first and second metatarsal bones in the depression distal to the junction of the bases of the two bones and over the dorsalis pedis artery
SP10^a^ (*Xuehai*)	With the knee flexed, on the medial side of the thigh 2 *cun* above the superior medial corner of the patella on the prominence of the medial head of the quadriceps muscle of the thigh
PC6^a^ (*Neiguan*)	On the medial aspect of the forearm between the palmaris longus and the flexor carpi radialis tendons, 2 *cun* proximal to the palmar wrist crease
DU20 (*Baihui*)	On the head at the anterior median line, 5 *cun* superior to the anterior hairline

Based on pattern and phase differentiation, syndrome 1 = Spleen–kidney Yang deficiency; syndrome 2 = Liver–kidney Yin deficiency, phase 1 = follicular phase; phase 2 = ovulatory phase

^a^Bilateral points

#### Manual acupuncture group

Disposable, stainless steel needles (0.25 × 40 mm, provided by Wuxi Jiajian Medical Instrument Co. Ltd., China) will be punctured through the device when patients are in the supine position and inserted 10–30 mm deep, depending on location. The needles will be retained for 30 min and manually lifted, thrust, twisted, and rotated moderately every 10 min to maintain the *deqi* sensation. When qi arrives, the acupuncturist will feel tightness around the needle, the patients will feel such sensations as soreness, numbness, distention, and heaviness at the point. These sensations can radiate along the course of the channel.

#### Sham acupuncture group

The procedure and duration of treatment in the SA group will be identical in the TA group except the needles (0.25 × 25 mm, blunt tip, provided by Wuxi Jiajian Medical Instrument Co. Ltd., China) are blunt tip and there will be no skin penetration or manual stimulation.

#### Practitioner background

The TA and SA treatments will be conducted by acupuncturists who are licensed by the Chinese medicine practitioner qualification certificate and who specialize in acupuncture with at least three years of clinic experience. They will have studied acupuncture for > 10 years and graduated from a TCM university. All practitioners will undergo intensive and customized training for a full understanding of the SA procedure and will be trained to administer acupuncture using a sham needle device. The techniques for the entire treatment procedure will be standardized between practitioners.

### Outcome measures

Outcomes will be collected at baseline, each menstruation, after three menstrual cycles of treatment, and follow-up at six months after the completion of the treatment. The overview of the outcome measurement at the different time points is shown in Table 3.

**Table 3:** Overview of study visits

	Screening and baseline visit	Menstrual cycles			Six months after the last acupuncture treatment
		1st	2nd	3rd	
General condition ^a^	√				
Gynecological examination	√				
Fasting blood samples for sex hormone ^b^, FINS, FBG, HbA1c	√			√	
Transvaginal ultrasound	√	√	√	√	
Pregnancy test (blood β-HCG)				√	
Treatment record ^c^		√	√	√	
Questionnaire ^d^	√			√	
Telephone follow-up ^e^					√

^a^General condition include age, BMI, menstrual and obstetrical histories

^b^Sex hormones include: E2, T, P, FSH, LH, PRL, DHEAS, SHBG

^c^Treatment record includes date and adverse events

^d^Questionnaire includes the PCOS and TCM syndrome scores

^e^Inquires if the participants were pregnant

*E2* estradiol, *T* total testosterone, *P* progesterone, *FSH* follicle-stimulating hormone, *LH* luteinizing hormone, *PRL* prolactin, *DHEAS* dehydroepiandrosterone sulfate, *SHBG* sex hormone-binding globulin, *BMI* body mass index, *FINS* fasting insulin, *FBG* fasting blood-glucose, *HbA1c* glycated hemoglobin

#### Primary outcome

The primary outcome is the pregnancy rate during the trial, including ectopic pregnancy. Blood β-HCG will be measured at 14 days after ovulation.

#### Secondary outcome

##### Ovulation rate

Ovulation will be observed through Basal body temperature (BBT) or B ultrasound every menstrual cycle. From the 10th day of the menstrual period, the patients will receive B ultrasound 3–4 times to observe the size of the follicle and whether it has discharged.

##### Syndrome scores

The PCOS syndrome scores will be recorded before and after treatment to assess whether the treatment improved the patient’s signs of PCOS.

The TCM syndrome scores will be recorded by the same professor once a week to adjust the acupuncture and herbal treatment [28] and evaluate the improvement of the patients’ TCM syndromes.

##### Blinding assessment

Assessment for patients’ blinding was conducted after the last treatment session. All patients will be asked to guess whether they have received TA or SA.

##### Sex hormones and IRI

The fasting blood samples will be drawing in the third day of menstruation before and after treatment by the nurse. The chemiluminescence immunoassay (CLIA) will be used to determine serum sex hormone levels include estradiol (E2), T, progesterone (P), FSH, LH, prolactin (PRL), dehydroepiandrosterone sulfate (DHEAS), sex hormone-binding globulin (SHBG), and fasting serum insulin (Model: Switzerland ELECSYS2011). The oxidase method will be used to measure fasting plasma glucose through automatic biochemical analyzer (Model: Japan OLMPUS AU 400). These tests will all performed by the Shuguang Hospital Medical Testing Center.

The gold standard for investigating and quantifying insulin resistance is the “hyperinsulinemic euglycemic clamp” [29], but it rarely performed in clinical because of the invasive. The insulin resistance index (HOMA-IR) is a simpler test and is calculated by the equation (HOMA-IR = fasting blood glucose × fasting insulin/22.5).

##### Follow-up

Six months after the completion of the treatment, follow-up will be performed by telephone to ask if the participants were pregnant.

### Adverse events/serious adverse events

We will monitor adverse events for each treatment during the trial, including hematomas and acute pain. Any adverse events or reactions that are thought to be causally associated with the intervention will be recorded, managed, and reported to the study coordinators. Serious adverse reactions will be reported to the ethical committee.

### Data analysis

All statistical analyses of the data will be performed using SPSS program V.21.0 (SPSS Inc., Chicago, IL, USA). A *P* value < 0.05 will be considered statistically significant. For cases in which we do not finish all the treatment, we select intention-to-treat (ITT) analysis to avoid the effects of crossover and drop-out. Measured data will be expressed as mean ± SD ($\overline{x}$ ±s) if it obeys a normal distribution or approximate normal distribution. Median (interquartile range [IQR]) will be used if the data do not obey the normal distribution and count data will be expressed in terms of the number of cases. We will use chi-square tests for categorical data and two-sample t-test or Wilcoxon rank sum test for continuous data, according to whether the data are normally distributed. The variance analysis will be performed for the difference between the two groups and within the group. The stratified analysis will be performed to control the confounding factor if necessary. Data analysis will be conducted by statisticians who are independent of the research team.

## Discussion

Acupuncture therapy is a major component of TCM and is increasingly widely used because it is non-invasive and has fewer side effects [30–32]. The drugs for ovulation induction may lead to adverse reactions such as OHSS and multiple pregnancy. Compared with previous studies, we combined the TCM therapy with scientific and rigorous experimental design to explore the effect of MA on infertile women with PCOS. The selection of acupoints and herbs is based on the syndrome differentiation and the period of the female menstrual cycle in order to adequately demonstrate the dialectical treatment of TCM.

In terms of the treatment period, the acupuncture treatment starts on the third day of menstruation to the third day of ovulation; therefore, acupuncture can be fully utilized to promote the development and discharge of follicles and can avoid the risk if the patient is pregnant.

The evidence for long-term efficacy of acupuncture in patients with PCOS is not sufficient. However, a neurological study [33] showed that though SA and placebo were as effective as TA in terms of reduction of symptoms and objective physiological outcomes, verum acupuncture was superior to SA in producing improvements in both peripheral and brain neurophysiological outcomes. Pregnancy is the ultimate indicator of infertility treatment and insulin resistance (IR) is a pathological condition in which cells fail to respond normally to the insulin [34–36]. Of people with PCOS, 50–80% may have IR at some level [37]. IR is closely related to the pathogenesis of PCOS [38]. Therefore, we select the pregnancy rate, ovulation rate, sex hormones, and IRI as indicators of efficiency of the trial. We expect to discover that MA can be more helpful for promoting follicular development, adjusting menstrual cycle, and increasing pregnancy rate in infertile women with PCOS.

## Trial status

At the time of initial manuscript submission, recruitment had already started (23 December 2017), but it has not been completed. The last patient is expected to be included in the study in February 2019.

## Additional file

Additional file 1: SPIRIT 2013 Checklist: Recommended items to address in a clinical trial protocol and related documents. (PDF 130 kb)

## Data Availability

The data that support the findings of this study will be available from authors but restrictions apply to the availability of these data, which will be used under license for the current study, and so are not publicly available. Data will be available from the authors upon reasonable request.

## Abbreviations

CAM: Complementary and Alternative Medicine
HOMA-IR: Insulin resistance index
IR: Insulin resistance
IRI: Insulin Resistance Index
OHSS: Ovarian hyper-stimulation syndrome
PCOS: Polycystic ovary syndrome
RCT: Randomized controlled trial
SA: Sham acupuncture
TA: True acupuncture
TCM: Traditional Chinese Medicine
